# The Development of Magnolol-Loaded Intravenous Emulsion with Low Hepatotoxic Potential

**DOI:** 10.3390/ph16091262

**Published:** 2023-09-06

**Authors:** Aleksandra Gostyńska, Joanna Czerniel, Joanna Kuźmińska, Izabela Żółnowska, Jakub Brzozowski, Violetta Krajka-Kuźniak, Maciej Stawny

**Affiliations:** 1 Department of Pharmaceutical Chemistry, Poznan University of Medical Sciences, 6 Grunwaldzka, 60-780 Poznan, Poland; jczerniel@ump.edu.pl (J.C.); jkuzminska@ump.edu.pl (J.K.); izabela.zolnowska@student.ump.edu.pl (I.Ż.); 80847@student.ump.edu.pl (J.B.); mstawny@ump.edu.pl (M.S.); 2 Department of Pharmaceutical Biochemistry, Poznan University of Medical Sciences, 4 Swiecickiego, 60-781 Poznan, Poland; vkrajka@ump.edu.pl

**Keywords:** magnolol, parenteral nutrition, liver disease, Box–Behnken design

## Abstract

Intestinal failure-associated liver disease (IFALD) is a severe liver injury occurring due to factors related to intestinal failure and parenteral nutrition administration. Different approaches are studied to reduce the risk or ameliorate the course of IFALD, including providing omega-3 fatty acids instead of soybean oil-based lipid emulsion or administering active compounds that exert a hepatoprotective effect. This study aimed to develop, optimize, and characterize magnolol-loaded intravenous lipid emulsion for parenteral nutrition. The preformulation studies allowed for chosen oils mixture of the highest capacity of magnolol solubilization. Then, magnolol-loaded SMOFlipid was developed using the passive incorporation method. The Box–Behnken design and response surface methodology were used to optimize the entrapment efficiency. The optimal formulation was subjected to short-term stress tests, and its effect on normal human liver cells and erythrocytes was determined using the MTT and hemolysis tests, respectively. The optimized magnolol-loaded SMOFlipid was characterized by the mean droplet diameter of 327.6 ± 2.9 nm with a polydispersity index of 0.12 ± 0.02 and zeta potential of −32.8 ± 1.2 mV. The entrapment efficiency of magnolol was above 98%, and pH and osmolality were sufficient for intravenous administration. The magnolol-loaded SMOFlipid samples showed a significantly lower toxic effect than bare SMOFlipid in the same concentration on THLE-2 cells, and revealed an acceptable hemolytic effect of 8.3%. The developed formulation was characterized by satisfactory stability. The in vitro studies showed the reduced cytotoxic effect of MAG-SMOF applied in high concentrations compared to bare SMOFlipid and the non-hemolytic effect on human blood cells. The magnolol-loaded SMOFlipid is promising for further development of hepatoprotective lipid emulsion for parenteral nutrition.

## 1. Introduction

Parenteral nutrition (PN) is a life-saving procedure conducted by intravenous route and implemented in the case of severe gastrointestinal tract disorders manifested by malnutrition and the inability to obtain the proper nutritional state using the enteral route. The indications for such therapy include severe intestinal inflammations in the course of exacerbation of Crohn’s disease or radiation enteritis, short bowel syndrome, profusely secreting gastrointestinal fistulas, gastrointestinal obstruction resulting from postoperative adhesions, or a neoplastic process, and other states causing a malabsorption syndrome. PN is also often used in conditions of digestive tract injuries, severe acute pancreatitis, severe burns, extreme exhaustion, and in the perioperative period and intensive care patients. Such therapy may be periodic (e.g., immediately after surgery) or lifelong (e.g., in patients with congenital necrotizing enterocolitis) [1,2]. PN provides all the nutrients necessary for the proper functioning of the body: water, amino acids, glucose, lipids, electrolytes, vitamins, and trace elements [3]. Besides its beneficial role for patients, PN is associated with the risk of acute and chronic complications, including cholestasis and hepatic steatosis. PN-associated liver disease is also known as intestinal failure-associated liver disease (IFALD). IFALD is a severe liver injury occurring due to factors related to intestinal failure and PN administration, with no other evident cause. The prevalence of IFALD can be as high as 85% in the case of home PN-dependent patients, and it is more often diagnosed in children than adults [4]. The incidence in children in all age groups is estimated at 49.8% [5], and in preterm infants receiving prolonged PN exceeding 90 days, it may even reach 90% [6].

The etiology of IFALD is multifactorial. Provision of excess calories in the form of glucose, along with lipids administered above 1 g/kg body weight, and intravenous administration of phytosterols in lipid emulsion rich in soybean oil are thought to increase the risk of such complications [7]. Different approaches are studied to reduce the risk or ameliorate the course of IFALD, including providing fish-oil-based intravenous lipid emulsions instead of those containing soybean oil or administering active compounds that exert a hepatoprotective effect. The limitation of the first method is the concern for essential fatty acid deficiency. Chronic reduction in the soybean oil-based lipid emulsion administration seems to outweigh the benefits. Soybean oil-based lipid emulsions contain an essential fatty acid which deficiency is particularly undesirable in the preterm infant due to possible impairment in brain growth and development [8]. Therefore, the second approach involving searching for active compounds that may reduce the problem of IFALD, including cholestasis and hepatic steatosis in PN-depended patients, attracts more and more attention. 

Magnolol is a polyphenolic active compound found in plants belonging to the genus Magnolia. It can be isolated from Magnolia officinalis bark. Due to its pleiotropic biological effects, including anti-inflammatory and antioxidant properties, extracts containing this compound have been used in traditional Chinese and Japanese medicine. The role of magnolol in preventing or treating IFALD has not been investigated so far. However, this compound is known to act as a hepatoprotective agent in alcohol [9] and high-fat-diet-induced [10,11,12] liver damage. Liu et al. [9] demonstrated that employing magnolol in a mouse model of alcoholic liver damage led to a significant reduction in the severity of the hepatic injury, including a decrease in hepatocyte necrosis and a reduction in liver damage markers, ALT and AST.

**Figure 1:**
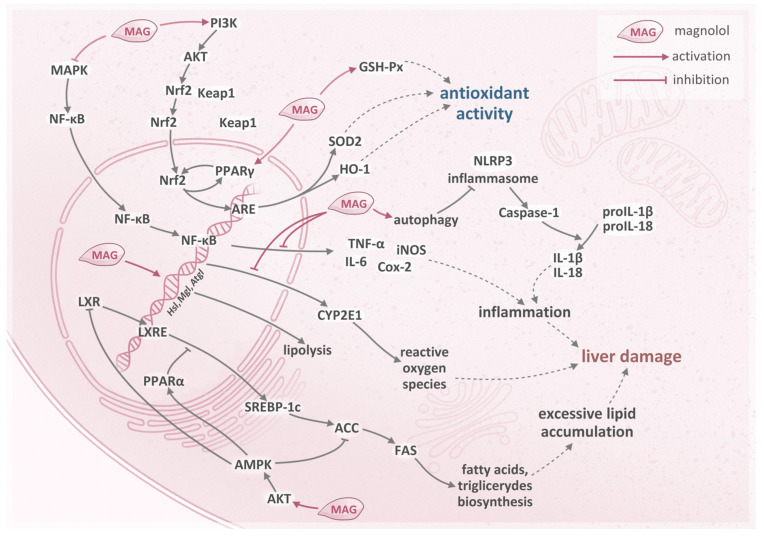
The mechanism of hepatoprotective action of magnolol. PI3K—phosphoinositide-3-kinase; AKT—AKT kinase; Nrf2—nuclear factor erythroid 2-related factor 2; Keap1—kelch-like ECH associated protein 1; PPARγ—peroxisome proliferator-activated receptor gamma; ARE—antioxidant response element; SOD2—superoxide dismutase 2; HO-1—heme oxygenase 1; GSH-Px—glutathione peroxidase; MAPK—mitogen-activated protein kinase; NF-κB—nuclear factor kappa B; TNF-α—tumor necrosis factor alpha; iNOS—inducible nitric oxide synthase; IL-6—interleukin-6; COX-2—cyclo-oxygenase-2; NLRP3—nucleotide-binding domain, leucine-rich–containing family, pyrin domain–containing-3; proIL-1β—prointerleukin-1β; proIL-18—prointerleukin-18; IL-1β—interleukin-1β; IL-18—interleukin-18; CYP2E1—cytochrome P450 2E1; Hsl—hormone-sensitive lipase; Mgl—monoacylglycerol lipase; Atgl—adipose triglyceride lipase; LXR—liver X receptor; LXRE—LXR response element; SREBP-1c—sterol-regulatory element binding protein-1c; ACC—acetyl-CoA carboxylase; FAS—fatty acid synthase; AMPK—adenosine monophosphate (AMP)-activated protein kinase; PPARα—peroxisome proliferator-activated receptor alpha (based on [9,10,12]).

Moreover, the effects of magnolol in a non-alcoholic fatty liver disease model were described, employing steatotic HepG2 cells and tyloxapol-induced hyperlipidemia mice/rats in the studies. Treatment with magnolol resulted in a decline in triglyceride levels and the maintenance of lipid homeostasis [10,12]. The utilization of magnolol also reversed the autophagy disturbances induced by lipotoxicity and provided antioxidative action—the level of hepatic superoxide anion was reduced [10]. The mechanisms underlying the protective effects of magnolol in hepatocytes are illustrated in Figure 1. 

Magnolol decreases inflammation by direct down-regulation of the expression of genes encoding pro-inflammatory cytokines and through suppression of the MAPK/NF-κB and NLRP3 pathways, which induces the formation of pro-inflammatory cytokines, including TNF-α, IL-6, and IL-18. Moreover, magnolol reduces the expression of enzymes such as iNOS and COX-2, and promotes the up-regulation of autophagy-related proteins ATG5-12, ATG7, Beclin1, and LC3BII/LC3BI ratio, which also regulates the inflammation process in the liver. The second mechanism involves the regulation of antioxidative pathways. Magnolol activates the Nrf2/HO-1 pathway by induction of the phosphorylation of PI3K and AKT, and stimulation of PPARγ. It also enhances the levels of antioxidative enzymes such as GSH-Px and SOD, and reduces the expression of CYP2E1. Magnolol is known to regulate lipid biosynthesis by decreasing the levels of SREBP-1c and FAS in the lipid biosynthesis pathway. This effect is achieved by activating the AKT/AMPK/PPARα pathway, consequently preventing liver steatosis. It is also known to directly modulate the expression of genes related to lipolysis and lipogenesis. All those mechanisms are responsible for decreasing lipid overload, oxidative stress, and inflammation in the liver [9,10,12]. 

Magnolol is a lipophilic compound with low water solubility and is, therefore, low in vivo bioavailability, limiting its application for oral drug delivery. The intravenous administration of magnolol to patients receiving PN is necessary since using the oral or enteral route is impossible or not recommended. Moreover, the parenteral administration allows for a reproducible pharmacokinetic/efficacy profile and high bioavailability due to bypassing the oral absorption barrier. However, the intravenous formulation must meet specific criteria to ensure safety, efficacy, and quality. Intravenous dosage forms must be sterile, apyrogenic, and meet the European Pharmacopeia (Ph. Eur.) requirements, such as >90% label claim, related substance level lower than the toxic qualified level, appropriate pH, and osmolality (OSM), and no particulate matter presence. Further, if the resulting dosage form is an intravenous emulsion, the mean droplet diameter (MDD) cannot exceed 500 nm, and should not reveal a hemolytic effect [13]. Therefore, this study was aimed at developing and optimizing the magnolol-loaded intravenous emulsion intended for PN and investigated its potential application in therapy.

## 2. Results

**Figure 2:**
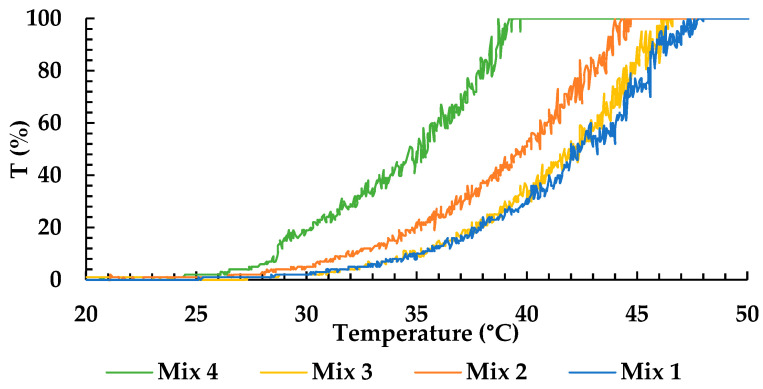
Solubility of magnolol in oils mixtures.

The solubility test was performed using the multi-reactor crystallizer to determine the intravenous emulsion with the highest solubilization capacity for magnolol. Crystal 16TM equipment detects the turbidity of the solution using a laser that passes through the vials. Moreover, this equipment works in temperature-increase-over-time mode, providing information on the solubility of selected substances in the chosen solvent with increased temperature. The higher transmittance of the solution at a lower temperature, the higher the solubility of the drug in the oil. Among the emulsions selected for this study, 100% transmittance in the lowest temperature was observed for oils composition found in SMOFlipid (Figure 2), and this emulsion was chosen to prepare magnolol-loaded emulsion (MAG-SMOF).

The preparation process of MAG-SMOF was optimized using the Box–Behnken design (BBD) methodology. This study involved the previously developed low-energy method [14]. The three-level, three-factor BBD model was employed, where the concentration of magnolol (X1), shaking speed (X2), and time of shaking (X3) were selected as independent factors. Table 1 shows the variables and their levels.

A total of 15 formulations, with three central points, were prepared and characterized (Table 2). The concentration of magnolol in MAG-SMOF was determined using the UV-Vis spectrometry method, and parameters such as EE% and DL% were calculated (Table 3).

**Figure 3:**
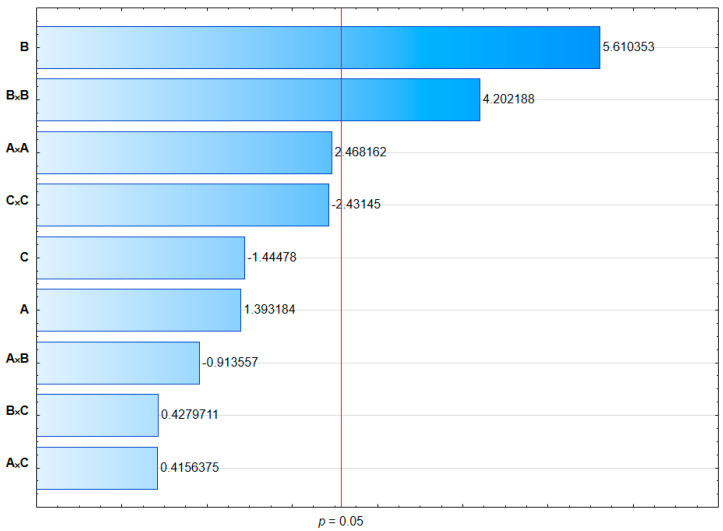
Pareto chart for entrapment efficiency of magnolol in MAG-SMOF formulations.

Results of the ANOVA test (Table 4) and analysis of the Pareto plot (Figure 3) allowed us to conclude that the model F-value of 7.51 implied the model for EE% was significant. There was only a 1.95% chance that an F-value could occur due to noise, and a *p*-value less than 0.05 indicated that model terms were significant. In this case, the shaking time turned out to be a significant model factor. The lack of fit F-value of 2.40 implied the lack of fit was not significant relative to the pure error, and there was an over 30% chance that a lack of fit F-value this large could occur due to noise. Response surface methodology was based on adjusting the curved surface determined in experimental tests, allowing for analysis of the obtained results. 

**Figure 4:**
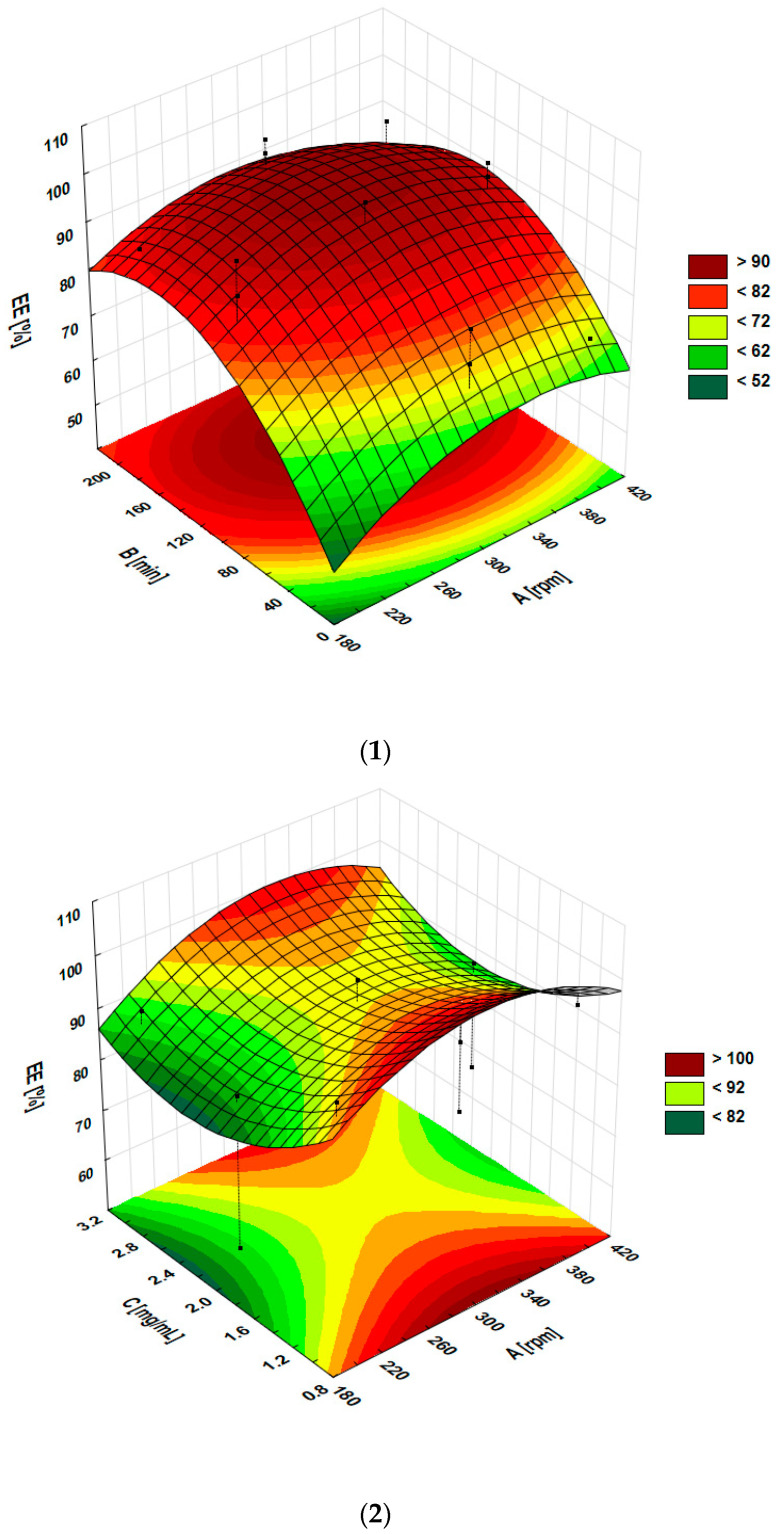

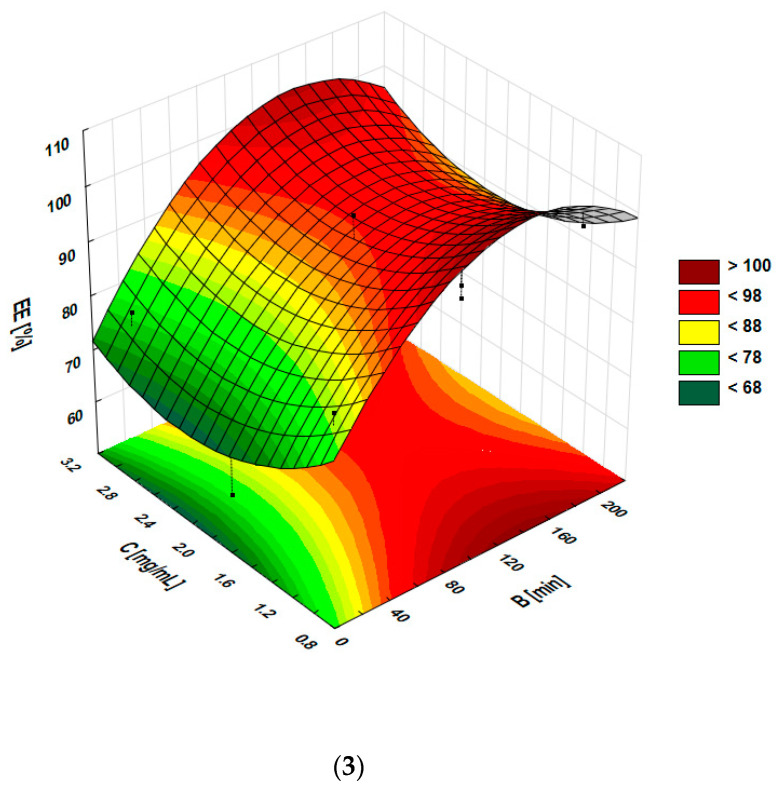
Response surface plots present the interaction effect of (**1**) time of shaking (B) and shaking speed (A), (**2**) concentration of magnolol (C) and shaking speed (A), and (**3**) concentration of magnolol (C) and time of shaking (B) entrapment efficiency.

The values of optimal parameters can be determined based on the shape of the response surface and, thus, effectively limit the number of experiments. The three-dimensional graphs presented in Figure 4 allow for assessing the effect of significant model terms on EE%. Three-dimensional graphs showed that the EE% depended on the shaking time, which was highest after about 120 min. Analysis of the charts showed the impact of magnolol concentration and shaking speed on EE% showed that the highest EE% was observed for the lowest and the highest concentrations when shaking speed was in the middle range of 300 to 350 rpm. Comparing these graphs, it can be seen that the impact of time of shaking is more significant than the impact of shaking speed on EE%. 

**Figure 5:**
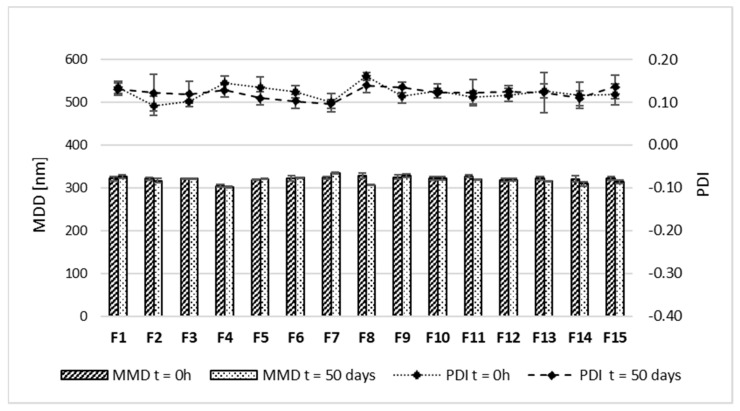
Results of MDD and PDI of MAG-SMOF immediately after preparation (t = 0 h) and after 50 days of storage (t = 50 days) at 4 ± 1 °C without light access. The left axis corresponds to MDD, and the right one to PDI.

**Figure 6:**
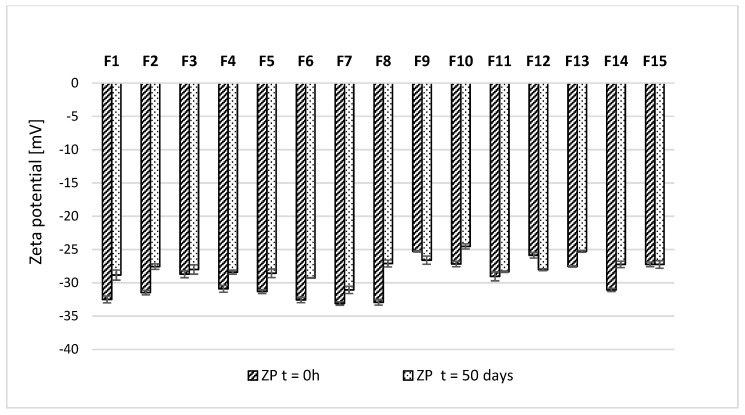
Results of ZP of MAG-SMOF immediately after preparation (t = 0 h) and after 50 days of storage (t = 50 days) at 4 ± 1 °C without light access.

Moreover, to determine the stability of lipid emulsion in MAG-SMOF formulations MDD, PDI, and ZP were measured immediately after preparation and after 50 days of storage at 4 ± 1 °C without light access (Figure 5 and Figure 6). SMOFlipid was characterized by MDD equal to 334.6 ± 2.4 nm with PDI 0.09 ± 0.05. The addition of magnolol to SMOFlipid led to a decrease in MDD. The MDD of MAG-SMOF was in the range of 304.5 ± 2.5–328.4 ± 5.9 nm with PDI ranging from 0.09 to 0.16, and no changes higher than 5% of the initial value were observed. The only exception was formulation F8, where the MDD decreased after 50 days of storage by 6.6%. 

The ZP corresponding to the electric potential at the slipping plane of micelles showed variability at the tested measurement points (Figure 6). However, the initial values obtained immediately after preparation were in the range of −25.3 ± 0.1–−33.1 ± 0.3 mV. After 50 days of storage, the ZP value increased to a maximum of 5.8 mV. The decrease in ZP value was detected only in the case of formulations F9 and F12, with a maximum difference between the first and 50 days of 2.2 mV. To evaluate the impact of magnolol on the stability of intravenous lipid emulsion, the same parameter was determined for bare emulsion without the addition of magnolol. The ZP of bare SMOFliplid was equal to −32.1 ± 0.3; thus, in most cases, the addition of magnolol slightly decreased the absolute values of ZP.

The BBD allowed for estimating the optimal process parameters, and the following parameters were applied to obtain the best formulation of MAG-SMOF: shaking speed of 358.9 rpm, time of shaking of 120.6 min, and concentration of magnolol equal to 2.97 mg/mL. The theoretical EE% for samples prepared with the assumption of such parameters was 98.22%, and the desirability value was 1.000. The optimal formulation (MAG-SMOF) was prepared and characterized by MDD equal to 327.6 ± 2.9 nm, PDI equal to 0.12 ± 0.02, and ZP equal to −32.8 ± 1.2 mV. The pH and OSM were equal to 7.19 and 401 mOsm/kg H_2_O, respectively.

**Figure 7:**
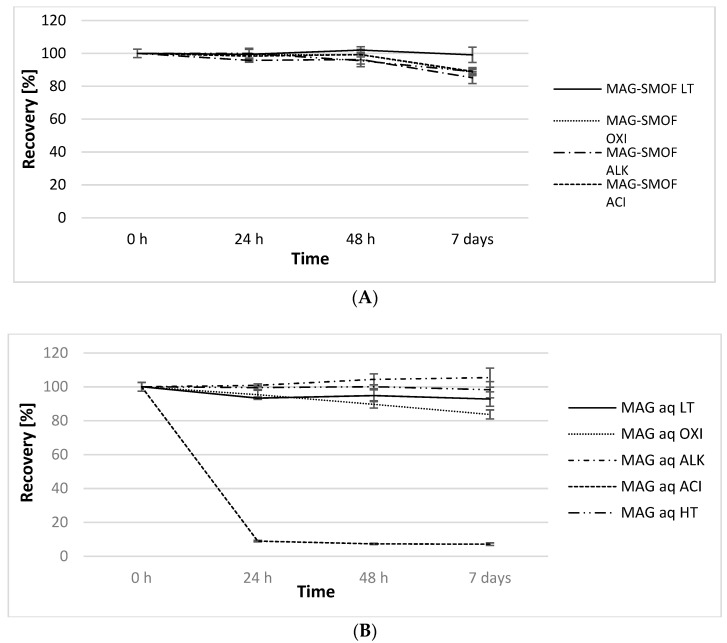
Results of MAG-SMOF (**A**) and MAG_aq_ (**B**) recovery during storage for 7 days exposed to various stress factors.

Stress tests were performed to assess the effect of different stress conditions, including oxidative stress (OXI), alkaline pH (ALK), acidic pH (ACI), and high temperature (HT) on the chemical stability of magnolol in MAG-SMOF. The potential protective effect of lipid emulsion on the stability of magnolol was evaluated by comparing results obtained for MAG-SMOF with MAGaq exposed to the same stress factors (Figure 7A,B). As a control, MAG-SMOF was stored at 4 ± 2 °C light-protected (low-temperature conditions, LT).

Magnolol was stable within 95% in all studied conditions for 48 h. However, after 7 days of storage, only samples stored in LT conditions pose satisfying concentration, i.e., over 90% of the initial value (99.10 ± 4.63%). In the case of MAGaq, the highest degradation of magnolol was observed in samples stored in ACI conditions. Its concentration dropped after 24 h to 8.96 ± 0.39% of the initial value, and after 7 days of storage was equal to 7.16 ± 0.74%. Moreover, after 48 h, MAGaq samples stored in OXI conditions also degraded over the pharmacopeial limit of 10%, reaching 89.64 ± 2.17% of the initial value.

**Figure 8:**
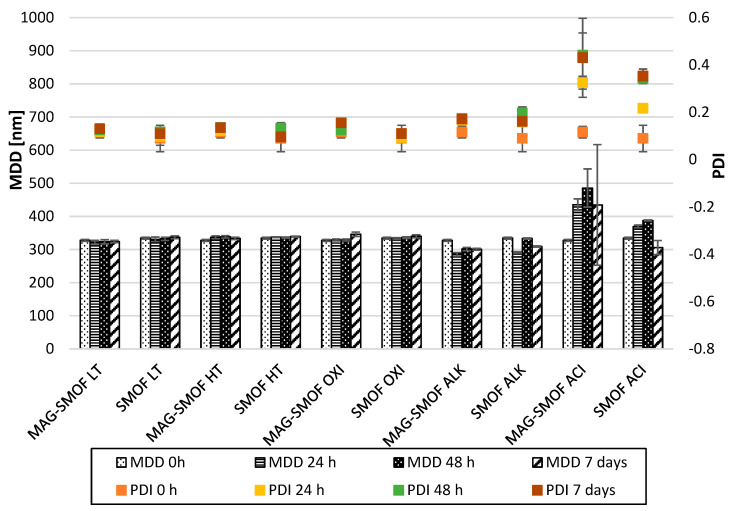
Results of MDD of magnolol-loaded SMOFlipid (MAG-SMOF) and bare SMOFlipid (SMOF) during storage for 7 days exposed to various stress factors. The left axis corresponds to MDD and the right one to PDI.

Additionally, the stability of the oil-in-water system under the stress conditions was evaluated by measuring the MDD and PDI of MAG-SMOF samples and bare SMOFlipid (Figure 8) and determining changes in other physicochemical parameters such as pH, OSM, and ZP of MAG-SMOF during storage (Table 5). The applied stress conditions, excluding extreme pH, did not significantly affect the MDD and PDI of lipid emulsion. In the case of ALK conditions, MAG-SMOF and SMOFlipid samples showed a decrease in MDD value within the first 24 h of storage, accompanied by the appearance of the second fraction of lipid emulsion droplets above 1000 nm.

Similarly, in the case of samples stored in ACI conditions, the second fraction of lipid droplets above 3000 and 2000 nm was detected for MAG-SMOF and SMOFlipid, respectively. ACI conditions affect the size of lipid emulsion, leading to the increase in MDD value up to 485.22 ± 57.84 nm for MAG-SMOF after 48 h. The high PDI values, which ranged from 0.33 to 0.44 and 0.22 to 0.35 for MAG-SMOF and SMOFlipid, supported the lack of homogeneity of samples stored in such conditions. In contrast, for samples stored in other conditions, the PDI was below 0.2 regardless of the presence of magnolol in the formulation.

Over the storage period, the pH, OSM, and ZP were measured. Results showed that the pH of MAG-SMOF samples affected by the stress factor excluding HT conditions did not change significantly over the 7 days of storage. The pH changes were of maximum ±0.2. In contrast, a 50% decrease in the pH value was observed in the case of MAG-SMOF samples stored in HT conditions. The OSM of samples after stress tests did not differ more than 5% from the initial value. Only in the case of samples under the OXI conditions the OSM could not be determined by the freezing point measurement method. The ZP changes were the most evident in extreme pH conditions. In samples stored under ALK conditions, the absolute value of ZP decreased in contrast to samples stored under ACI conditions, where the absolute value of ZP increased.

To determine injectability, the assessment of the possibility of administering the developed magnolol-loaded SMOFlipid formulation through different needle sizes using an infusion syringe pump was performed. As a comparator, the reference intravenous lipid emulsion (SMOFlipid) and water for injections were used. The results of the injectability test are presented in Table 6. 

It was shown that the addition of magnolol to commercial lipid emulsion did not affect the injectability of the emulsion. Both MAG-SMOF and SMOFlipid were possible to infuse using a syringe 21 G or bigger with all infusion rates selected for this study. In the case of smaller needles, the higher infusion rate limited the possibility of administration. In the case of needle 27 G, the infusion was impossible even when an infusion rate of 25 mL/h was applied. The inversely proportional dependence of the increase in pressure on the increase in the diameter of the needle used, and the increase in the infusion rate was also confirmed for the water for injection used as a control. However, in this case, infusion was possible to perform at much higher rates and smaller needle sizes (continuous infusion was possible to perform with the rate of 100 mL/h through all applied needles).

**Figure 9:**
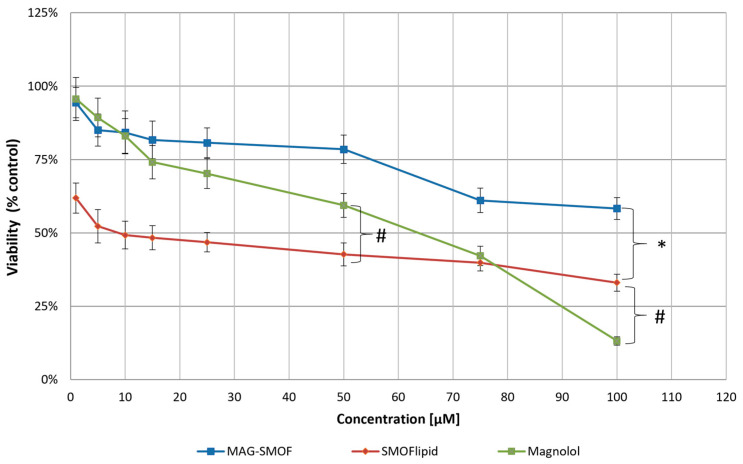
The effect of the magnolol, SMOFlipid, and MAG-SMOF on the viability of THLE-2 cells after 24 h incubation. The cell viability was measured by MTT assay. A cell culture medium was used as a control. Data are expressed as the mean ± SEM from three separate experiments. Dunnett’s multiple comparison test assessed statistical significance between groups (* *p* < 0.05 as the comparison between SMOFlipid and MAG-SMOF; # *p* < 0.05 as the comparison between SMOFlipid and magnolol).

MTT assays were used to evaluate the impact of magnolol, SMOFlipid, and MAG-SMOF on the viability of THLE-2 cells. A high concentration of emulsion was applied to investigate the potential protective effect of magnolol. Samples containing magnolol were prepared in the 5–100 µM concentration range. The dilution of SMOFlipid in the same manner as MAG-SMOF samples allowed the comparison of the toxic effect of lipid emulsion on the cells. In investigated concentrations, SMOFlipid reduced the viability of normal liver cells. The toxic effect of magnolol was lower than SMOFlipid in concentrations ranging from 5 to 75 µM and surpassed its toxic effect in the highest concentration. However, the MAG-SMOF samples showed a significantly lower toxic effect than bare SMOFlipid in the same concentration (Figure 9). 

The hemolysis test was performed to evaluate the safety of intravenous administration of the developed formulation. For this purpose, MAG-SMOF was incubated with red blood cells at 37 °C, and its hemolytic effect was evaluated in comparison to positive and negative controls. Additionally, the influence of SMOFlipid on erythrocytes was assessed. Results showed that SMOFlipid and MAG-SMOF revealed an acceptable hemolytic effect of 6.3% and 8.3%, respectively.

## 3. Discussion

Intravenous lipid emulsions have already been used in clinical practice as a drug-delivery system, allowing for intravenous administration of poorly water-soluble substances, including propofol, etomidate, diazepam, and fat-soluble vitamins [15,16]. Moreover, many substances loaded into lipid emulsion are under investigation and intended for oral [17,18,19], intranasal [20], or intravenous [14,17,21] administration. Among those studies, commercially available PN lipid emulsions were also used. Intravenous lipid emulsions are a crucial ingredient of PN. They are a source of non-protein energy and essential fatty acids. Therefore, its composition is clinically relevant [22,23]. Administration of soybean oil-based intravenous lipid emulsion, which has a high content of polyunsaturated fatty acids and phytosterols, is known to impair liver function, increase the infection and sepsis risk, and is associated with a prolonged hospital stay. On the other hand, using lipid emulsion containing omega-3 fatty acids improves markers of liver function, triglyceride levels, and shortens the length of hospital stay. Moreover, such emulsions, due to the presence of animal-derived oil, namely, fish oil, contain a lower concentration of phytosterols and are known to be less likely to cause IFALD than other soybean oil-based lipid emulsions.

Currently, there are many clinical trials on IFALD. Most focus on applying intravenous lipid emulsion containing fish oil only (Omegaven) to prevent cholestasis in parenteral nutrition-fed patients. The main concern about using this emulsion is a potential deficiency of essential fatty acids. Interestingly, among studies concerning other solutions aimed at reducing IFALD, only four substances used as a therapeutic agent can be found: vitamin D, taurine, L-carnitine, and NST-6179 (orally administered, fully synthetic medium-chain fatty acid analog). For this reason, searching for new substances of therapeutic potential in IFALD seems to be an interesting approach.

Since the composition of commercial intravenous lipid emulsion’s oil phase varies, its solubilization capacity may differ. Therefore, this work performed a preformulation test to assess the solubility of magnolol in oil or oil mixtures found in the most popular commercial fat emulsions. Crystal 16^TM^ combines automation with integrated transmissivity technology, which is a convenient method allowing the screening of a large number of liquid lipids in a short time using a low amount of drug substances and without the use of an organic solvent [24]. Comparing oil mixtures selected for this study, corresponding to commercially available intravenous lipid emulsions, Mix 4 composed of 30% of soybean oil, 30% of MCT, 25% of olive oil, and 15% of fish oil, possessed the best solubilization capacity [25] for magnolol. Mix 4 is distinguished from other oil mixtures by the presence of olive oil, which is rich in oleic acid (over 70% of fatty acids) [26]. Other studies also show a good solubilization capacity of this fatty acid. The solubility of nebivolol in pure oleic acid was about 30 mg/mL compared to 5 mg/mL for soybean oil, which contains about 25% of oleic acid and about 50% of linoleic acid [26].

Based on preformulation studies, we chose SMOFlipid, a commercial lipid emulsion of a known safety profile used for PN. The standard dose of SMOFlipid is 1.0–2.0 g fat/kg body weight/day, corresponding to 5–10 mL/kg body weight/day. Compared to soybean oil-based lipid emulsions, the mixed oils lipid emulsions contain fewer phytosterols and are characterized by a better fatty acids profile. Therefore, they are considered advisable in the case of IFALD prevention or treatment [27], although clinical studies in this area are ambiguous. Yu et al. showed that the incidence and development of cholestasis in surgical neonates receiving an intravenous mixed-oil lipid emulsion is even higher than in the case of soybean oil-based lipid emulsion. However, the mix-oils lipid emulsion was characterized by a significantly shorter latency to developing cholestasis. The authors concluded that up to date, there is still no intravenous lipid emulsion able to prevent IFALD [28]. On the other hand, Daniel et al. showed that in pediatric patients, administration of SMOFlipid was associated with a lower prevalence of IFALD than in patients receiving soybean oil-based lipid emulsion [29]. 

This paper aimed to add magnolol to SMOFlipid. The process of the active substance incorporation was optimized to obtain formulation with the highest EE%. To limit the number of formulations prepared the Box–Behnken methodology was applied. The results showed that using the shaking speed of 358.9 rpm, time of shaking of 120.6 min, and concentration of magnolol equal to 2.97 mg/mL, the highest EE% equal to 98.22% can be achieved. In our previous studies concerned with the honokiol-loaded Lipofundin MCT/LCT, we used the same independent variables and found that 5 factors significantly influenced the EE% of honokiol. In the case of MAG-SMOF, the EE% was significantly influenced by only one factor, i.e., time of shaking. This suggests that the solubilization capacity of magnolol in SMOFlipid was better than honokiol in Lipofundin. Considering the similar lipophilicity of honokiol and magnolol (logP = 4.5) [30], the influence of the oil-phase composition of those lipid emulsions seems crucial on the solubility of these substances. 

All obtained formulations in the optimization process were subject to the long-term stability studies of lipid emulsion. Since intravenous lipid emulsions are colloidal formulations, their physiochemical stability may be affected by adding other substances or the technological process used to incorporate them into the oil-in-water system. Different studies showed that commercial intravenous lipid emulsions are quite resistant to various technological processes, including bath sonication [31], horn sonication [32], or high-pressure homogenization [33]. Therefore, the impact of shaking using a horizontal shaker applied in our study, known as a passive incorporation method, was considered a low risk of causing loss of emulsion stability. Nevertheless, we investigated if the incorporation of magnolol may affect lipid emulsion stability. After 50 days of storage, the results showed that all magnolol-loaded formulation was characterized by the MDD within the pharmacopeial limit of 500 nm and low PDI, which indicates the homogeneity of the lipid emulsion. The MDD is a critical safety parameter in the context of intravenous administration. The USP set the limit for MDD determined using the DLS method, which cannot exceed the value of 500 nm [34] since the administration of bigger droplets may lead to catheter occlusion and liver capillaries’ embolization [13]. Another important factor characterizing intravenous lipid emulsion is ZP. After 50 days of storage, the ZP values fluctuated, with a decrease in their absolute values in most cases. Such results may suggest the destabilization potential of magnolol. However, no correlation between observed ZP and magnolol concentration was found, and all studied formulations were characterized by sufficient absolute value of ZP to consider the developed formulation as physically stable. 

For optimal magnolol-loaded SMOFlipid formulation, further short-term stress studies were performed. Intravenous lipid emulsions are prone to destabilization processes caused by various factors. Adding other substances, pH changes, temperature, and oxidative stress may affect the physical stability of lipid emulsion and the chemical stability of the added active substance. On the other hand, incorporating the active substance into the lipid emulsion system may result in a protective effect on the active substances, or the addition of a polyphenolic compound due to its antioxidant ability may defend the lipid peroxidation [35]. Short-term stress tests showed SMOFlipid could protect magnolol against ACI and OXI stress degradation. Significant differences between magnolol recovery in the MAGaq and MAG-SMOF samples were observed in those conditions. The ability to protect active substances against oxidative stress may be explained by the presence of all-rac-α-tokoferol in SMOFlipid, which is an antioxidant added to pharmaceutical formulations to protect fatty acid peroxidation. However, the highest degradation was observed for MAGaq in ACI conditions, with recovery below 10% after the first 24 h. This condition also affected lipid emulsion stability, leading to increased MDD and PDI values and the appearance of the second fraction of lipid emulsion droplets with a diameter above 1000 nm in MAG-SMOF and SMOFlipid. Since the droplet size of the second fraction in the case of SMOFlipid was smaller than that observed in MAG-SMOF samples, the MDD values of SMOFlipid were less obvious. MDD is evaluated as intensity-weighted MDD, which means the measure of the intensity fluctuations of the light scattered by the particle is performed during its evaluation. Therefore, not only the size but also the number of large droplets are counted. Additionally, it is considered that this method gives greater weighting to the large particles than the volume-weighted distribution. The second fraction of lipid droplets was also detected for lipid emulsion exposed to high pH. However, the results of MDD and PDI indicated lower destabilization potential compared to ACI conditions since, in this case, the homogeneity of the samples remained high. It should be noted, however, that the stress tests were conducted under extreme pH conditions of about pH = 13 for ALK conditions and about pH = 1 for ACI conditions. On the other hand, for tests conducted under other stress conditions, i.e., exposure to oxidative stress and high temperature, the obtained magnolol-loaded SMOFlipid formulation showed sufficient physicochemical stability of the lipid emulsion with MDD values that did not exceed the USP limit. 

The rheological characteristics of developed formulations in the form of oil-in-water emulsion determine not only their behavior during intravenous administration but also could have an impact on the safety upon administration and limit their use as drug dosage forms [36]. USP or Ph. Eur. Pharmacopoeias do not provide any specific measurement techniques and requirements to allow for injectability testing, therefore, we decided to use the infusion syringe pump equipped with the occlusion pressure alarm to compare the pressure exerted during the infusion of the developed formulation with a reference intravenous lipid emulsion (SMOFlipid), an authorized medicinal product. Different needle sizes and infusion rates were used to evaluate the differences between studied formulations. We are aware that the needle size determines the maximum infusion rate, and the applied rates, especially in the case of needles with small diameters (25 G, 26 G, and 27 G), were not in line with their clinical use.

Nevertheless, only high infusion rates were allowed to achieve a pressure alarm, the minimum programmable value of 75 mmHg. Such a system allowed us to compare the injectability of both formulations. The obtained results enabled us to state that the rheological characteristic of SMOFlipid was not affected by the addition of the magnolol since the injectability of MAG-SMOF and SMOFlipid was the same. 

Finally, the optimal formulation was the objective of in vitro assays and the hemolysis test. The MTT studies were performed to evaluate the ability of magnolol-loaded formulations to prevent the toxicity of intravenous lipid emulsion. For this reason, high concentrations of SMOFlipid were applied to reveal the toxic effect on THLE-2 cells. Its effect was compared with magnolol and MAG-SMOF. The results showed that adding magnolol to SMOFlipid decreases its toxic effect on normal liver cells, showing a hepatoprotective potential of magnolol-loaded SMOFlipid. The hemolysis tests were conducted to evaluate the impact of developed intravenous lipid emulsion on the red blood cells and to determine the safety of intravenous administration of such formulation. Adopting 10% as the acceptance criteria from other authors [37], our results indicate that the developed formulation can be administered intravenously for further in vivo studies.

Total parenteral nutrition-dependent patients are exposed to deficiencies of exogenous substances of plant origin known as natural bioactive compounds such as polyphenols, flavonoids, and anthocyanins, which, although not considered essential, are characterized by health-promoting properties. Although no flavonoid deficiency disease has been identified, long-term administration of bioactive compounds-depleted nutrition could lead to chronic persistent inflammation [38]. It is already known that patients with different conditions may require higher than standard doses of vitamins [39,40] and other natural compounds [41] found in parenteral nutrition, and such modifications are widely studied. Therefore, adding magnolol to parenteral nutrition lipid emulsion is an important step toward modifying parenteral nutrition admixture and supplementing it with bioactive compounds, which may positively affect the health of the parenteral nutrition-depended patients.

## 4. Limitations

The presented research has some limitations. First is the lack of the sterility of the developed formulation, which necessitates further studies before administering the drug intravenously to animals or humans. Such studies may involve three available sterilization methods: filtration, thermal, or radiation [42,43]. The first of them, due to the droplet size of the lipid emulsion, which exceeds the pore diameter of the sterilization filter, may be problematic to use. The next ones will require determining their impact on the active substance and the emulsion itself. In the case of radiation sterilization, the studies will involve sterilization of the magnolol in the substance and introducing its sterile form into intravenous lipid emulsion in an aseptic process. Therefore, the effect of the sterilized magnolol on the formation of reactive oxygen species in the lipid emulsion should be assessed. 

The clinical application of such formulation will require in-depth in vivo studies followed by clinical trials on its safety and efficiency. Before introducing such formulation into clinical practice, it is also crucial to perform long-term stability studies (shelf-life tests) and check the compatibility with other ingredients of parenteral nutrition admixtures. Since such data allow for successful implementation and immediate practical application of the developed formulation [13,44]. Nevertheless, as shown in many other studies on intravenous lipid emulsion, the sterilization, and long-term stability studies are not always presented at such an early stage of the formulation development [45]. Despite those limitations, the presented results showed great potential for magnolol-loaded intravenous lipid emulsion and may contribute to changing the approach to treating IFALD and focusing on the natural bioactive compounds deficiency in parenteral nutrition-depended patients. 

## 5. Materials and Methods

### 5.1. Materials

Magnolol was purchased from Pol-Aura, Poland, and SMOFlipid was purchased from Fresenius Kabi AB, Sweden. Purified soybean oil 700 (Ph. Eur., USP), purified olive oil (Ph. Eur., USP), purified fish oil (Ph. Eur. Type I), and MCT (Ph. Eur., USP) were very kindly gifted by Lipoid GmbH (Ludwigshafen, Germany). Water for injection was purchased from B. Braun Melsungen AG, Melsungen, Germany. All organic solvents used in the studies were of analytical or high-performance liquid chromatographic grade. Epithelial human liver cells, THLE-2 (CRL-2706), were purchased from the American Type Culture Collection (ATCC), Manassas, VA, USA.

### 5.2. Methods 

#### 5.2.1. Selection of PN Emulsions

To select commercial PN emulsions with the highest solubilization capacity for magnolol, pre-formulation studies were performed. The qualitative and quantitative composition of the oil phase of commercial PN emulsions selected for this study is presented in Table 7. To determine the solubilization properties, 500 mg of the mixtures of oils that correspond to the composition of the oil phase of four commercial PN emulsions, i.e., Intralipid (Mix 1), Lipofundin MCT/LCT 20% (Mix 2), Lipidem (Mix 3), and SMOFlipid (Mix 4), were prepared in 2 mL vials. 

Then, 40 mg of magnolol was added to each vial and was subject to the analysis using Crystal 16™ (Crystal Pharmatech Inc., Cranbury, NJ, USA). The solubility of magnolol in oil mixtures was determined by the measurements of the turbidity of the sample expressed as % of transmittance in a function of time with the gradually increasing temperature. The following parameters were used: bottom stirrer speed of 700 rpm, a heating and cooling ramp of 2.7 °C/min, and a maximum temperature of 80 °C. In samples presenting 100% transmittance, magnolol was fully solubilized.

#### 5.2.2. Optimization of the Preparation Process of Magnolol-Loaded PN Emulsion

Magnolol-loaded PN emulsions were obtained by horizontal shaking of magnolol with SMOFlipid. The applied low-energy method involved horizontal shaking using GLF 3005 horizontal. Briefly, 10, 20, or 30 mg of magnolol was weighed into the 12 mL glass tubes, filled with 10 mL of SMOFlipid, and shaken. After the incorporation process, samples were collected, kept for 24 h at 4 ± 1 °C to let unincorporated substance sink to the bottom, and filtered through a 0.45 µm cellulose filter. The Box–Behnken design and response surface methodology were used to optimize the preparation process. The influence of three independent variables (magnolol concentration, shaking speed, and time of shaking) in three levels coded as −1, 0, and 1 on the loading efficiency (LE%) of magnolol in PN emulsion was investigated. The assumed levels of each independent variable are presented in Table 1. 

Based on the Box–Behnken design, a total of 15 experiments with three focal points were designed (Table 2). The optimization process results were subjected to regression analysis using the StatSoft package of Statistica 13.1 software (StatSoft Polska Sp. z o.o. Krakow, Poland). During the analysis, a 2-factor model was chosen. The data were analyzed using the Pareto plot, the correlation of the approximated versus observed value, the response surface plot, and a regression equation determination.

#### 5.2.3. Determination of Physicochemical Parameters

##### Determination of pH

The pH was measured using a SevenCompact pH-meter (Mettler Toledo, Columbus, OH, USA). Before using the equipment, the calibration with buffer solutions of pH 4.00 ± 0.05, pH 7.00 ± 0, and pH 10.00 ± 0.05 was performed.

##### Determination of Osmolarity

The OSM was determined using an Osmometer 800 CLG (Tridentmed, Warszawa, Poland) by the freezing point measurement method. Before its use, the instrument was calibrated with the standardizing solution (0 mOsm/kgH_2_O, cat. no. 800.02) provided by the device supplier. For each measurement, 100 µL of undiluted samples were transferred to dedicated microtubes and placed into the measuring head.

##### Determination of Droplet Size

The dynamic light scattering (DLS) method was used to determine the droplet size of the lipid emulsion. Measurements were performed using Zetasizer Nano ZS (Malvern Instruments, Worcester UK) equipped with a 633 nm laser at a fixed scattering angle of 173°. The detection cell temperature was kept constant at 25 °C. Samples for analysis were diluted 1:100 with water for injection, transferred to a dedicated polycarbonate cuvette, and placed in a measuring cell. Measurements were performed in triplicates, and the results were expressed as polydispersity index (PDI) and MDD.

##### Evaluation of Zeta Potential

To determine zeta potential (ZP), the laser Doppler electrophoresis (LDE) technique was used. Measurements of samples diluted 100 times with water for injection were performed in triplicate using the Zetasizer Nano ZS (Malvern Instruments, Worcester, UK). Measuring cell temperature was kept constant at 25 °C. The ZP was determined based on the electrophoretic mobility of the micelles and calculated using the Smoluchowski equation.

##### Determination of Magnolol Concentration in MAG-SMOF Using Spectrophotometry UV-Vis

Magnolol concentration in MAG-SMOF samples was determined using the UV-Vis spectrophotometry method at room temperature on a Lambda 20 UV/Vis Spectrophotometer (PerkinElmer, Shelton, CT, USA). To collect the UV–Vis absorption spectra, 100 µL of MAG-SMOF was dissolved in 1 mL of dichloromethane, and the sample was filled up to 10 mL with methanol. Each sample was transferred to 1.0 cm quartz cells, and the measurements were conducted with respect to the intensity of light passing through a blank sample consisting of 100 µL of SMOFlipid (without the addition of magnolol), 1 mL of dichloromethane and filled up to 10 mL with methanol. The concentration of magnolol was calculated based on calibration curve performer in the range of 0.004 to 0.044 mg/mL with absorption determined at 291.2 nm. The calibration curve was performed by the dilution of the appropriate volume of standard stock solutions of magnolol in methanol (1 mg/mL) in a 10 mL of standard diluent obtained by the binary mixture of dichloromethane and methanol (1:9) and the addition of 100 µL of SMOFlipid. 

##### Determination of Magnolol Concentration in MAG-SMOF Using HPLC-FLD Method

The new HPLC method for determining magnolol concentration in injectable lipid emulsions has been developed and validated. The chromatographic analysis of MAG-SMOF was performed on an Agilent 1260 Infinity II LC system (Agilent Technologies, Waldbronn, Germany) equipped with fluorescence detectors (FLD). The detection wavelengths were adjusted at 293 nm (extinction) and 370 nm (emission). The separation was performed on the reverse phase column (C-18(2) 100 Å Luna, 150 × 4.6 mm ID, 5 µm, 211 Phenomenex, Torrance, CA, USA, and isocratic solvent system of acetic acid 0.4% (21%), acetonitrile (25%), and methanol (54%) were used as mobile phase. The injection volume was 10 µL, and the time analysis was 15 min. The concentration of magnolol was calculated based on the calibration curve, which was performed in the range of 0.75 to 22.5 µg/mL. The standard stock solution of magnolol was obtained by dissolving 30 mg of magnolol in 10 mL of methanol (3 mg/mL). The working solutions for the calibration curve were obtained by diluting the appropriate volume of standard solution in 10 mL of the diluent consisting of the binary mixture of dichloromethane and methanol (1:9) and adding 100 µL o SMOFlipid. The method was validated according to the International Council for Harmonisation Requirements (ICH), and the method’s linearity, precision, and accuracy were determined.

#### 5.2.4. Calculation of Entrapment Efficiency and Drug Loading of Magnolol in MAG-SMOF

The entrapment efficiency (EE%) and drug loading (DL%) of magnolol in MAG-SMOF were determined by directly measuring magnolol concentration in obtained formulations using the UV-Vis spectrophotometry method. Before the measurement, samples were filtered through the 0.45 µm cellulose filter to separate unincorporated substances from the formulation. 

The EE% (Equation (1)) of magnolol in MAG-SMOF was calculated using the following equation:

(1) $$EE\%=\frac{Amount\;of\;magnolol\;entrapped}{Total\;amount\;of\;magnolol\;added\;to\;parenteral\;nutrition\;nanoemulsion}\times 100\%$$

The DL% (Equation (2)) of magnolol in MAG-SMOF was calculated using the following equation:

(2) $$DL\%=\frac{Amount\;of\;magnolol\;entrapped}{Amount\;of\;oil\;phase\;of\;SMOFlipid+Amount\;of\;magnolol\;entrapped}\times 100\%$$

It is reported that the solubility of magnolol in water is very low, <1 mg/mL; therefore, for the calculation of EE% and DL%, the magnolol content in the water phase was ignored.

#### 5.2.5. Mid-Term Stability Tests 

The stability studies were performed to evaluate the effect of the addition of magnolol on the physical stability of lipid emulsion. The stability studies of MAG-SMOF were performed for all formulations obtained during the optimization process (Box–Behnken design). Samples were stored at 4 ± 1 °C without light access. The MDD, PDI, and ZP were determined after preparation and 50 days of storage. All measurements were performed in triplicate, and the results are shown as mean value ± standard deviation. 

#### 5.2.6. Short-Term Stress Tests of Optimized MAG-SMOF Formulation

The short-term stress tests were performed to assess the stability of magnolol and lipid emulsion in different stress conditions and to evaluate the potential protective effect of obtained formulation on magnolol stability. For this purpose, the stability studies were performed for the optimal formulation of MAG-SMOF and compared with results obtained for 3 mg/mL magnolol aqueous solution (MAGaq), where the aqueous phase consists of 50% water for injection, 49.9% absolute ethanol, and 1% Tween 80. The MAG-SMOF and MAGaq stored at 4 ± 1 °C were used as controls. 

The thermal stress test was performed to assess the stability of magnolol at high temperatures. For this purpose, 2 mL of MAG-SMOF and MAGaq was transferred to 6 mL sterile glass vials, capped, and placed into a thermostatic chamber with the temperature set at 80 ± 1 °C. To investigate the influence of oxidative stress on the magnolol stability 1 mL of sample (MAG-SMOF or MAG aq) was combined with 1 mL of 30% H_2_O_2_ in 6 mL sterile glass vials, capped, and stored at the temperature of 20 ± 2 °C without exposure to the light. To investigate the influence of pH on the magnolol stability 1 mL of sample (MAG-SMOF or MAG aq) was combined with 1 mL of 0.5 M NaOH or 0.5 M HCl in 6 mL sterile glass vials, capped, and stored at the temperature of 20 ± 2 °C without exposure to the light. 

In predetermined intervals (t = 0, 24, 48, and 168 h), the concentration of magnolol was determined using the HPLC-FLD method. Additionally, pH, OSM, MDD, and ZP of MAG-SMOF were evaluated to assess the stability of lipid emulsion in the same intervals. All samples were prepared in triplicates.

#### 5.2.7. Injectability Test

To determine the injectability of the developed formulation, we assessed and compared the pressure exerted by the MAG-SMOF and the reference lipid emulsion (SMOFlipid) when administered through different needles using an infusion syringe pump (Perfusor compact plus, B Braun Melsungen AG, Malsugen Germany). As a control, we used water for injection. Perfusor Compact Plus is equipped with a pressure sensor that detects an occlusion. The higher the pressure level is set, the higher the pressure level must rise before triggering an occlusion pressure alarm. In this study, the pressure limit was set to 75 mmHg, which means that if the pressure applied to the drug injected exceeds this value, an alarm will be triggered. To perform this study 30 mL of the samples were drawn into a 50 mL syringe (original Perfusor syringe) and were infused with different infusion rates (25, 50, 75, 100, or 200 mL/h and using the bolus mode with the rate of 800 mL/h) through different needles (27 G, 26 G, 25 G, 23 G, 22 G, 21 G, 20 G, and 18 G). The occurrence of an alarm within 1 min of administration was recorded. The test was conducted in three replicates.

#### 5.2.8. In Vitro Cytotoxicity Studies

Epithelial human liver cells, THLE-2 (CRL-2706, ATCC, Manassas, VA, USA), were maintained in the bronchial epithelial growth medium (BEGM) supplemented with the Bullet Kit (Lonza, Cologne, Germany) and, additionally, in 10% FBS, 5 ng/mL epidermal growth factor, and 70 ng/mL phosphoethanolamine, and cultured at 37 °C in humidified 5% CO_2_ atmosphere. The MTT assay assessed the effect of magnolol, SMOFlipid, and MAG-SMOF on cell viability according to the standard protocol. Briefly, THLE-2 cells (10^4^/well) were seeded in a 96-well plate. After 24 h of preincubation in a complete medium, the tested emulsions were added to the culture medium at 1 to 100 µM concentrations. The cells were incubated for a further 24 h. After incubation, the cells were washed with phosphate-buffered saline (PBS), and a fresh complete medium containing the MTT salt (0.5 mg/mL) was added and incubated again for 4 h. In the last step, formazan crystals were dissolved in isopropanol containing HCl, and the absorbance was detected at 540 and 690 nm using the plate reader TECAN Infinite M200. The statistical analysis of the obtained results was performed using the GraphPad Instat 3 version. To assess the significance of the differences in the evaluated parameters, one-way ANOVA with Dunnett’s post-test was performed with a significance level of *p* < 0.05.

#### 5.2.9. Hemolysis Assay

The hemolysis assay was performed to evaluate the safety of the intravenous administration of MAG-SMOF. Briefly, the red blood cells were obtained from a healthy person group A female with normal blood chemistry. The erythrocytes were suspended in PBS with pH 7.4, and the washing step was repeated three times. In the next step, according to the published procedure by Jumaa et al. [46], 2 mL of the erythrocytes suspension was incubated for 5 min with 2 µL of 0.9% saline (positive control) or Triton^®^ X-100 (negative control), MAG-SMOF, or SMOFlipid. After incubation, the mixtures were spun in a centrifuge at 200× *g* for 10 min. The supernatants were used to detect the amount of hemoglobin released by Drabkin’s reagent [47]. The hemolytic potential of nanoemulsions was expressed as % hemolysis and calculated via Equation (3):

(3) $$\%\;hemolysis=\frac{A_{Sal}-A_{S}}{A_{Sal}-A_{TRI}}\times 100\%$$

where ASal is the absorbance value for samples containing 0.9% saline, AS is the absorbance for emulsion samples, and ATRI is the absorbance for samples containing Triton^®^ X-100 [37].

## 6. Conclusions

The presented study allowed for the development, optimization, and characterization of the MAG-SMOF intravenous lipid emulsion with hepatoprotective potential. The Box–Behnken design and response surface methodology allowed for the optimization of the formulation, leading to over 98% entrapment efficiency. The optimal formulation was characterized by physicochemical parameters sufficient for parenteral administration and satisfactory resistance to stress conditions. The in vitro studies showed the reduced cytotoxic effect of MAG-SMOF applied in high concentrations compared to bare SMOFlipid and the non-hemolytic effect on human blood cells.

## Figures and Tables

**Table 1:** Independent variables and their levels.

Independent Variables	Coded Levels		
	−1	0	1
Shaking speed (rpm) (A)	200	300	400
Time of shaking (min) (B)	15	105	195
Magnolol concentration (mg/mL) (C)	1	2	3

**Table 2:** Experiments designed using the Box–Behnken model.

Formulation Code	A Shaking Speed (rpm)	B Shaking Time (min)	C Magnolol Concentration (mg/mL)
F1	200	15	2
F2	400	15	2
F3	200	195	2
F4	400	195	2
F5	200	105	1
F6	400	105	1
F7	200	105	3
F8	400	105	3
F9	300	15	1
F10	300	195	1
F11	300	15	3
F12	300	195	3
F13	300	105	2
F14	300	105	2
F15	300	105	2

**Table 3:** Characteristics of studied MAG-SMOF formulations.

Formulation Code	EE% ± SD (%)	DL% ± SD (%)
F1	56.39 ± 4.56	0.58 ± 0.04
F2	70.97 ± 2.67	0.71 ± 0.02
F3	85.84 ± 3.05	0.85 ± 0.02
F4	91.24 ± 4.96	0.93 ± 0.04
F5	97.14 ± 1.78	0.49 ± 0.01
F6	94.97 ± 2.21	0.45 ± 0.01
F7	89.88 ± 3.43	1.34 ± 0.04
F8	91.89 ± 1.67	1.36 ± 0.02
F9	85.04 ± 6.75	0.44 ± 0.03
F10	97.93 ± 6.80	0.47 ± 0.03
F11	77.78 ± 1.66	1.19 ± 0.02
F12	94.98 ± 1.33	1.42 ± 0.02
F13	97.77 ± 2.21	0.98 ± 0.02
F14	90.78 ± 3.34	0.89 ± 0.03
F15	92.14 ± 2.47	0.95 ± 0.02

**Table 4:** Results of ANOVA test for entrapment efficiency of magnolol in MAG-SMOF formulations.

Source	Sum of Squares	df	Mean Square	F-Value	*p*-Value	*p*-Values
Model	1707.83	9	189.76	7.51	0.0195	<0.05
A	49.10	1	49.10	1.94	0.2221	>0.05
B	796.20	1	796.20	31.51	0.0025	<0.05
C	52.79	1	52.79	2.09	0.2080	>0.05
A × B	21.07	1	21.07	0.8338	0.4031	>0.05
A × C	4.37	1	4.37	0.1729	0.6948	>0.05
B × C	4.64	1	4.64	0.1838	0.6860	>0.05
A × A	153.99	1	153.99	6.09	0.0566	>0.05
B × B	446.40	1	446.40	17.67	0.0085	<0.05
C × C	149.57	1	149.57	5.92	0.0592	>0.05
Residual	126.34	5	25.27			
Lack of fit	98.87	3	32.96	2.40	0.3077	>0.05
Pure error	27.47	2	13.73			
Cor. total	1834.17	14				
Regression equation	EE% = 32.75214 + 0.418125 A + 0.448469 B − 32.41917 C − 0.000255 A × B + 0.010450 A × C + 0.011972 B × C − 0.000646 A × A − 0.001357 B × B + 6.36458 C × C					

**Table 5:** Physicochemical characterization of magnolol-loaded SMOFlipid (MAG-SMOF) during storage for 7 days exposed to various stress factors.

Sample	Conditions	pH (t = 0 h → t = 7 day)	OSM (mOsm/kg) (t = 0 h → t = 7 day)	ZP (mV) (t = 0 h → t = 7 day)
MAG-SMOF LT	4 ± 2 °C	7.19 → 7.18	401 → 394	−29.0 → −28.6
MAG-SMOF HT	80 ± 1 °C	7.17 → 3.56	394 → 411	−30.6 → −34.3
MAG-SMOF OXI	30% H_2_O_2_	4.50 → 4.64	-	−26.7 → −26.5
MAG-SMOF ALK	0.5 M NaOH	12.66 → 12.64	645 → 632	−69.3 → −59.3
MAG-SMOF ACI	0.5 M HCl	0.92 → 1.00	756 → 767	−8.4 → −18.4

**Table 6:** Results of the injectability test.

Sample Name	Infusion Rate (mL/h)	Needle Size (G)							
		27	26	25	23	22	21	20	18
MAG-SMOF	25	-	+	+	+	+	+	+	+
	50	-	-	-	-	+	+	+	+
	75	-	-	-	-	+	+	+	+
	100	-	-	-	-	-	+	+	+
	200	-	-	-	-	-	+	+	+
	800	-	-	-	-	-	+	+	+
SMOF	25	-	+	+	+	+	+	+	+
	50	-	-	-	-	+	+	+	+
	75	-	-	-	-	+	+	+	+
	100	-	-	-	-	-	+	+	+
	200	-	-	-	-	-	+	+	+
	800	-	-	-	-	-	+	+	+
Water for injection	25	+	+	+	+	+	+	+	+
	50	+	+	+	+	+	+	+	+
	75	+	+	+	+	+	+	+	+
	100	+	+	+	+	+	+	+	+
	200	-	-	+	+	+	+	+	+
	800	-	-	-	+	+	+	+	+

Key: - infusion impossible to perform (occlusion pressure alarm triggered), + continuous infusion possible to perform, MAG-SMOF—magnolol-loaded SMOFlipid, SMOF—SMOFlipid.

**Table 7:** The qualitative and quantitative composition of the oil phase of commercial PN emulsions.

Product Name	Soybean Oil	MCT	Olive Oil	Fish Oil
Intralipid	100%	-	-	-
Lipofundin 20% MCT/LCT	50%	50%	-	-
Lipidem	40%	50%	-	10%
SMOFlipid	30%	30%	25%	15%

## Data Availability

The data presented in this study are available on request from the authors.
